# Association of programmatic factors with low contraceptive prevalence rates in a rural area of Bangladesh

**DOI:** 10.1186/1742-4755-10-31

**Published:** 2013-06-19

**Authors:** Humayun Kabir, Nirod Chandra Saha, Elizabeth Oliveras, Rukhsana Gazi

**Affiliations:** 1 Centre for Equity and Health Systems, icddr,b, Mohakhali, Dhaka 1212, Bangladesh; 2 Health Systems and Infectious Diseases Division, icddr,b, Mohakhali, Dhaka 1212, Bangladesh

**Keywords:** Family planning, Programmatic factors, Contraceptives prevalence rate, Rural area, Bangladesh

## Abstract

**Objective:**

The study was conducted to identify selected programmatic factors relating to low contraceptive-use in a low-performing rural sub-district in Sylhet division of Bangladesh.

**Methods:**

A cross-sectional survey was carried out among 6983 currently-married women of reproductive age (MWRA) (15–49 years). To estimate the association between current contraceptive-use and other selected factors, multivariate analyse were performed, estimating the crude and adjusted odds ratios (OR), including 95% confidence intervals (CI).

**Results:**

The use of health facility by the MWRA in the last three months, distance from the residence to the nearest health facility, and contact with field workers in the last six months was significantly associated with contraceptive prevalence rate (CPR). There were potential differences regarding CPR, sources of contraceptive supply and Family Welfare Assistant (FWA) visit between hard to reach and non-hard to reach unions of Nabiganj sub-district.

**Conclusion:**

Strategies should be devised to increase the accessibility of MWRA to contraceptive methods by increased partnership with non-public sector and increased contacts with outreach workers through introducing community volunteers, and mobile phones help lines, by organizing frequent satellite clinics (SCs) and making community clinics (CCs) functional. Innovative strategies should be piloted for improving use of contraception in such hard to reach and low performing locality.

## Introduction

In 2000, more than 190 nations declared to achieve the Millennium Development Goals (MDGs) by 2015. The family Planning (FP) program addressed under MDG 5 is targeted to achieve desired family size, reduce total fertility, and slow population growth [1]. The use of modern contraceptive methods are increasing in the Eastern Europe and Central Asia but many less developed countries still face significant challenges in achieving desired family size, and reduce total fertility [2]. According to UN report, contraceptive prevalence rate varies widely between developed and developing countries [3]. For instance, low contraceptive prevalence rates are reported in Cambodia (24%) and Africa (27%) compared to United Kingdom (82%) and Spain (81%) [4,5]. Various studies have found programmatic factors to be associated with low contraceptive prevalence rates [6,7]. For example, the study done in Cambodia highlighted that outreach activities by FP field workers and accessibility to FP related information to married women of reproductive age were significantly associated with use of modern contraceptives [6].

The FP program of Bangladesh is considered as a success story in the field of population [8]. During the last 25 years, fertility among women of reproductive age in Bangladesh declined dramatically [9]. The decline was rapid from the mid-1970s, when the total fertility rate (TFR) was 6.3 children per women, to the early 1990s, when the TFR was 3.4 [10]. However, since the 1990s, the TFR has remained almost constant. Four successive Bangladesh Demographic and Health Survey (BDHS) showed a TFR of 3.4 in 1993–1994, 3.3 in 1996–1997, 3.3 in 1999–2000, and 3.0 in 2004 [10-13]. This plateau of TFR for over a decade has puzzled the nation’s policy makers and program implementers. In such a situation it is duly recognized to come up with evidence-based innovative strategies to encourage further decline of fertility in the country.

Available evidences indicate towards wide geographic variation within the country with high TFRs in Sylhet (3.7) and Chittagong (3.2) and low TFR in Khulna (2.0) and Rajshahi (2.6) [14]. This commensurate with the high contraceptive prevalence rate (CPR) in Rajshahi division (66%) & Khulna division (63%) and low CPR in Sylhet division (32%) & Chittagong division (44%) [14]. Investigating the reasons for such geographic variations in fertility rates might provide important clues to designing innovative strategies for increasing contraceptive use in low performing areas of Bangladesh. Previous studies have been identified a number of factors which may lead to low use of contraception. These include uneven distribution of population among FWAs (Government outreach workers who provide FP services in the form of motivation and distribution of oral pills and condoms, referral of clients for intrauterine device (IUD) and sterilization, in addition to other health services) [15,16], low motivation of the field workers, poor counseling on contraceptive methods [17], and inaccessibility of hard to reach areas for service provision [18]. Andaleeb et al. and others found that women are more likely to use contraception in areas with low population to FP worker ratios and when they receive more visits from the [19,20] workers. The average travel time from the fieldworker’s home to the client’s home is also associated with use of contraception [20]. Neaz et al. reported that 22% of FWAs contacted married women for FP services in densely and easily accessible areas compared to less than 10% in hard-to-reach areas [18]. All these findings add up to point towards the association of contacts with FP field workers with the level of contraception use by MWRA.

The low performance of the FP program is also attributable to poor supervision [20]. In addition, work planning, supervision, and performance-based rewards and punishment systems for FP workers are lacking where management systems are weak [19]. Such inadequacies are thought to limit the effective delivery of FP services in Bangladesh.

Nabiganj is a sub-district under Sylhet division that is a low performing Division [14]. Although the socio-cultural environment and indicators in Nabiganj sub-district are remarkably different from National indicators; the CPR is low, maternal and infant mortality rates are high, the TFR is high and health-seeking behavior is low, it represents many other low performing areas of Bangladesh. In Nabiganj, the population has increased by 15% in the last 10 years from 247,000 in 1991 to 285,000 in 2001 [21,22]. There were regional variations in educational attainments [14]; the highest percentage of women had never attended school in Sylhet division among six administrative divisions compared to Barisal Division. People living in Sylhet had second highest wealth quintiles (22.2%) among the six administrative divisions while Dhaka had the highest wealth quintile (28.0%). In Bangladesh, almost one in three households had a mobile telephone, while urban households were twice as likely to own a mobile telephone as rural households [14].

The highest percent of women of Chittagong and Sylhet divisions got married after legal age (18+ years) among six divisions [23]. In Bangladesh, the median age at first marriage for women aged 20–49 was 16 years [14]. Women in Khulna and Rajshahi had the lowest age at marriage [24].

In 2005–2008, the United Nations Population Fund (UNFPA), in collaboration with the National Institute of Population Research and Training (NIPORT) implemented the “Demand-based Reproductive Health Commodity Project (DBRHCP).” The goal of the project was to improve the capacity for increased access to and utilization of client-centred quality reproductive healthcare. The project was implemented in two low-performing sub-districts: Nabiganj in Habiganj district of Sylhet division and Raipur in Lashmipur district of Chittagong division**.** However, in the current investigation we were interested to explore the situation of a hard to reach area, so we purposively presented results from one area that is Nabiganj as the other area was not considered as hard to reach area. A household survey of MWRA was conducted in the project areas to obtain data for eventual assessment of programme impact. In an initial assessment for the DBRHCP, it was found that, in Nabiganj the coverage of population by the FWAs is low. While the Government recommends that each FWA covers a population of 5,000, in Nabiganj 52.5% of FWAs surveyed covered larger populations. Likewise, although each FWA is mandated to cover 450 eligible couples, only 6.6% of them do so. The majority (57.4%) of FWAs covers 451–900 couples, and 36% cover more than 900 couples. More than half of the unions (the smallest local government entities in Bangladesh) in Nabiganj are considered hard to reach.

Thus, understanding the key factors influencing contraceptive use in low-performing areas like Nabiganj can throw light on innovative policy and strategic decisions that Bangladesh can make further reduction in TFR.

### Rationale for the study

The Ministry of Health and Family Welfare (MoHFW), Government of Bangladesh (GoB), ranked districts and sub-districts based on four indicators using health and FP services utilization: percentage of pregnant women, crude birth rate, contraceptive-use rate, and coverage of two doses of vitamin A capsules. The MoHFW found that most low performing districts/sub-districts were located in Sylhet and Chittagong divisions [25]. Nabiganj is a low performing sub-district under Sylhet division. More than half of the areas are hard to reach. The present study was intended to explore programmatic factors related to low performance of FP program in hard to reach areas. Therefore we have selected Nabiganj sub-district purposively for this study based on the above mentioned criteria.

### Objective

The present study was intended to identify programmatic factors relating to the low contraceptive-use in a sub-district in Sylhet division of Bangladesh.

## Materials and methods

### Study design and population

We conducted a cross-sectional survey among 6983 MWRA (15 to 49 years) from November 2006 to March 2007 in Nabiganj sub-district of Sylhet division of Bangladesh. Eight unions out of twelve (the smallest local government rural units/entities in Bangladesh) in Nabiganj are considered hard to reach (remote geographical locations and slipping population from all sorts of development activities) [26]. Twenty four experienced female interviewers were collected data. Interviewers were supervised by three field research officers. Each day after returning from the field, the interviewers crosschecked the completed questionnaires. The field supervisors reviewed each of the questionnaires and conducted regular spot-checking to maintain data quality.

We listed every household and used systematic random sampling to select the MWRA. We considered the current rates of use of FP methods, antenatal care (ANC), and postnatal care (PNC) to estimate the required sample size at 95% confidence, with 95% power. We surveyed the MWRA on their use of FP methods, accessibility to reproductive health services, and use of SCs (an outreach activity – was introduced by government to deliver primary level of Maternal Child Health and Family Planning (MCH-FP) services by female paramedics which is being held 8 days in each month in each union in the rural areas commonly at the households of elite/elected representative of local government) using structured questionnaires.

### Analysis of data

We used the SPSS software (version 10) for determining potential associations between current contraceptive-use (Current contraceptive-use has been defined “in the BDHS survey 2007, as the proportion of currently married women who report that they are currently using a family planning method”) and programmatic factors (independent variables). The study covered following programmatic factors; contact with outreach workers have been defined as home visit by an FWA in the last six months, source of FP methods (public sector that includes public hospitals and centres, government satellite clinics, and field workers), NGO sector includes clinics run by NGOs and private sector includes pharmacy and other private clinics and practitioners, distance to the nearest health facility was assessed in two criteria (Within 1 km or more than 1 km). Crude and adjusted odds ratio were calculated with CI to assess association between CPR and associated programmatic factors. The statistical significance of differences in selected FP indicators between the survey and the BDHS 2007 was determined using two-sample comparison of proportions.

Multivariate logistic analyze were done to identify factors associated with contraceptive-use.

### Ethical consideration

Ethical approval for the study was obtained from the Ethical Review Committee of icddr,b. All participants gave their verbal consent to participate in the study. None refused to participate. To ensure confidentiality and anonymity, each participant was identified only by an identification number.

### Limitation of the study

A potential limitation of the present study was that we used the national survey data of the BDHS 2007 relating to rural areas to compare the selected indicators of family planning service with our survey data due to unavailability of sub-district-wise level indicators. Another limitation was that we used data from only one rural area. However, similar studies can be done in other low performing areas to confirm the results.

## Results

### Characteristics of MWRA

As expected, >50% of the MWRA in both the areas were aged 20 to 34 years (Table 1). However, fewer (6.6%) MWRA in Nabiganj were aged 15 to 19 years compared to 13.7% at the national level.

**Table 1:** Characteristics of MWRA in Nabiganj (2006) and in rural areas nationally (2007)

**Characteristics**	**Rural women nationally (N = 8514) %**	**Women in Nabiganj (N = 6983) %**
**Age (years)**		
15-19	13.7	6.6
20-24	19.7	17.2
25-29	17.3	19.7
30-34	15.0	16.8
35-39	13.6	14.7
40-44	10.9	12.9
45-49	9.6	12.1
**Education**		
No education	36.7	48.4
Primary incomplete	21.8	20.5
Primary complete	8.6	14.7
Secondary incomplete	23.9	13.5
Secondary complete and higher	8.9	2.8
**Household possessions**		
Wardrobe	37.0	38.3
Table	66.0	61.3
Chair	67.8	68.1
Watch	63.2	59.9
Radio	23.3	22.7
TV	21.9	29.6
Bicycle	27.9	7.8
Mobile phone	25.3	30.2

Multiple responses allowed.

The education levels were lower in Nabiganj than among rural women nationally. 48.4% of the MWRA in Nabiganj had never been attended school, while it was 36.7% nationally. Likewise, only 2.8% of the MWRA in Nabiganj had secondary or higher education compared to 8.9% in the national survey (BDHS 2007).

The percentage of households owning selected assets was almost similar in Nabiganj and Nationally. However, the households in Nabiganj were more likely to own television (TV) (29.6% vs 21.9%) and mobile phone (30.2% vs. 25.3%) than the national rates and less likely to own a bicycle (Table 1).

### Selected programmatic factors linked with family-planning services utilization

Less than 6% of the MWRA reported having been visited by an FWA in the past six months which is significantly less than 17% found in the national survey (BDHS 2007) (p < 0.005) (Table 2). Likewise, only 15% of the MWRA reported having visited a government satellite clinic for reproductive health services in the last six months compared to 32% in the national survey (BDHS 2007). Among those women who were modern contraceptive users, women in Nabiganj were less likely to have received their method from a government satellite clinic or FWA (42% vs 56%, p = 0.001) and more likely to have used private-sector services (52% vs. 34%, p = 0.001) (Table 2).

**Table 2:** Selected programmatic factors of family-planning services utilization: comparison between the national BDHS 2007 and survey of Nabiganj

**Programmatic factors**	**Rural women nationally (BDHS 2007) (N = 7909) (%)**	**Women in Nabiganj (N = 6983) (%)**	**P value**
**Modern contraceptive prevalence rate**	46.0	19.7	0.001
**MWRA who had ever:**			
Visited satellite clinic	32.0	15.3	0.001
Received government FWA visit within the last 6 months	17.2	5.7	0.001
**MWRA who had received contraceptive**	(n = 2784)	(n = 1230)	
Public sector	55.7	41.5	0.001
NGO sector	4.0	2.7	0.001
Private medical sector	33.5	51.9	0.001
Other private sectors	6.4	3.8	0.001

### Programmatic factors associated with contraceptive-use by MWRAs

Programmatic factors: As expected, women who had contacts with field workers in the last six months were more likely to be current contraceptive-users compared to women having no contacts (OR = 2.57; CI 1.98-3.34) (Table 3). The frequency of visit (one or more visits compared to no visit) to the MWRA by an FWA was associated with the use of contraception (OR = 1.71; CI 1.20-2.42) & (OR = 2.47; CI 1.72-3.56) respectively. Similarly women who reported having the nearest health facility within one kilometer were more likely to use contraceptive methods compared to others (OR = 1.41; CI 1.19-1.69). However, after adjusting for other indicators one associated variable became insignificant; for example relationship of frequency of contacts by household visits. Whereas, “use of health facility for any services in last 3 months” became significantly associated with CPR after adjustment.

**Table 3:** Programmatic factors associated with current contraceptive-use by MWRA

**Programmatic factors**	**Crude OR (95% CI)**	**Adjusted OR (95% CI)**
**Age (in years)**		
15-19	0.57 (0.40-0.82)*	0.39 (0.28-0.54)*
20-24	1.34 (1.08-1.66)	0.94 (0.81-1.11)
25-29	1.99 (1.63-2.42)*	1.43 (1.24-1.64)*
30-34	2.57 (2.11-3.14)*	1.72 (1.49-1.98)*
35-39	2.22 (1.81-2.74)*	1.35 (1.16-1.58)*
40+ (RC)	1	1
**Women’s education**		
No education (RC)	1	1
Primary level	1.65 (1.43-1.89)*	1.22 (1.09-1.38)*
Secondary level	2.50 (2.11-2.96)*	2.02 (1.76-2.33)*
**Contact with FWA in the last six months**		
No contact (RC)	1	1
Contact	2.57 (1.98-3.34)*	2.54 (2.11-3.05)*
**Frequency of contacts by household visits**		
No visit (RC)	1	1
1	1.71 (1.20-2.42)*	0.65 (0.42-1.00)
2 +	2.47 (1.72-3.56)*	0.94 (0.60-1.47)
**Type of nearest health facility**		
Health facility located more than1Km (RC)	1	1
Health facility within 1km	1.41 (1.19-1.69)*	1.42 (1.22-1.64)*
**Use of health facility for any services in the last 3 months**		
Non-use (RC))	1	1
Use	1.05 (0.92-1.20)	1.22 (1.07-1.39)*

*p<0. 001; **p<0. 005; *CI* = Confidence interval; *OR* = Odds ratio; RC-Reference category.

Non-programmatic factors: Non-programmatic factors, such as age and education were associated with the use of contraception.

### Programmatic factors of Family planning services utilization: difference between hard to reach and Non-hard to reach unions of Nabiganj

Modern contraceptive use rate was low in hard to reach unions (16%) compared to non-hard to reach unions (21%), a difference was 5%. Only 7% of MWRA in hard to reach unions received contraceptive from public sector, While 9% of MWRA in non-hard to reach unions received FP method from public sector. The contraceptive supply of private sector was 8% in hard to reach union compared to non-hard to reach unions 12%. Only 5% of MWRA received home visits by FWAs within last 6 months which was reported to be less than 7% in non-hard to reach unions (Table 4).

**Table 4:** Programmatic factors of Family planning services utilization: difference between hard to reach unions and Non-hard to reach unions of Nabiganj

**Programmatic factors**	**Hard to reach n = 4663%**	**Non-hard to reach n = 2320%**
Modern Contraceptive prevalence rate	16	21
Received contraceptive from Public sector	6.5	9
Received contraceptive from Private sector	8.3	11.8
Received FWA visit within last 6 months	4.8	7.4

### Contraceptive prevalence rate

**Figure 1:**
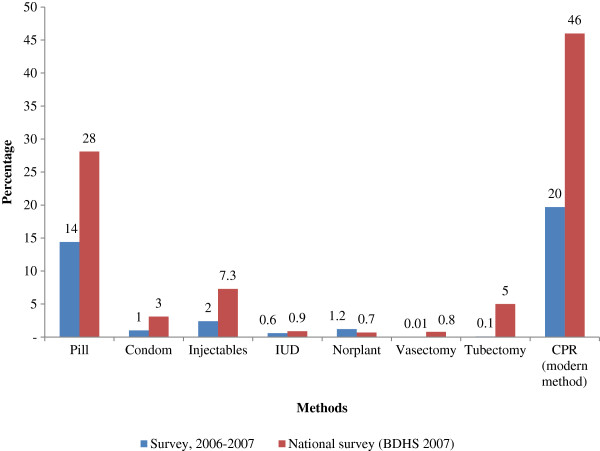
Comparison of contraceptive-use in Nabiganj (2006) and rural areas nationally (2007).

Only 1239 respondents (20%) of 6288 MWRA were currently using any modern contraceptive method, which is much lower than the national rate of 46% in rural areas (BDHS 2007). Among women surveyed, the use of all FP methods other than Norpalnt was lower than the national rates. The use of condom and injectable was more than three times lower, and pill-use (14%) was half the national rate (28%) (Figure 1).

### Contraceptive prevalence rate in hard-to-reach and non-hard-to-reach unions

**Figure 2:**
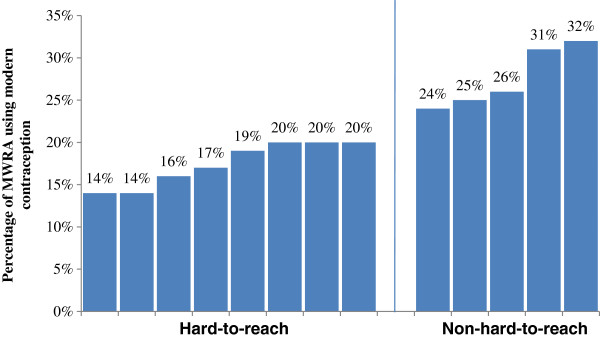
Comparison of contraceptive prevalence rates in hard-to-reach and non-hard-to-reach unions of Nabiganj sub district.

Within Nabiganj, the CPR also varied in hard to reach and non-hard to reach unions. It was particularly low in the hard-to-reach unions. A CPR of 14 to 20% was found in the hard-to-reach unions. While the CPR in the non-hard-to-reach unions ranged from 24 to 32% (Figure 2).

## Discussion and conclusions

The study intended to identify some of the important programmatic factors which are associated with low performance in contraceptive prevalence rates in the study area. The study highlighted that the low-level of client-worker contact and poor use of SCs are associated with the low level of contraceptive-use. There were potential differences regarding CPR, sources of contraceptive supply and FWA visit between hard to reach and non-hard to reach unions of Nabiganj sub-district. It is hope that the findings would assist the policy makers and program personnel adapting relevant programmatic modifications for better performance in the locality and similar low performing areas of Bangladesh.

The frequency of contact with outreach workers appeared to be one of the associated factors in increased use of contraceptives. While more than 10% of the FWAs in the study area had additional workload because they were compelled to cover multiple areas due to the shortage of staff [27]. However, ensuring contact with the outreach workers is found to be important as indicated by previous research [27] but it is difficult to achieve. Thus it is very crucial that recruitment process to fill up the vacant position of the outreach workers should be in a timely manner. The study on the Bangladesh Rural Advance Committee’s (brac) FP program in some low-performing sub-districts and hard-to-reach areas in Habiganj found that the shortage of field staff and staff drop-outs played a major role in the low use of contraception [28]. This study further reported that uneven distribution of work and the high worker-population ratio resulted in overload of work, which contributes to the low use of contraception. This study also recommended that qualification for outreach workers should be relaxed to fill-up all the vacant positions at field level in the hard-to-reach areas, and their working areas should be flexible to accommodate constraints. The MCH-FP Extension Project of International Centre for Diarrhoeal Diseases Research, Bangladesh (icddr,b) also highlighted that worker-population ratio was important [16,20] for programmatic achievements.

The present study highlighted geographical variations in the CPR when compared figures in the study area with national level indicators. The variations in the CPR also exist within the study area in hard to reach and non-hard to reach unions. A study done by brac reported the FWAs contacts (22%) with MWRA were found to be higher in non-hard-to-reach areas which were densely populated and easily accessible, while low contact (9.5%) by FWAs reported in hard-to-reach areas [18]. This study concluded that inaccessibility to the hard-to-reach areas might be associated with the low CPR. This study found that 42% of households were not visited by family welfare assistants where it is difficult to physically reach to the locality [18]. A study done in Cambodia found that women who were exposed to family planning information from mass media were more likely to use contraceptive than those who were not [6]. Moreover, the client-worker interaction is found to be another important factor improving the use of contraception [16] and program performance.

The use of SCs was also associated with use of contraceptive methods, meaning that the poor use might be associated with irregular SC sessions. The findings of a study in the Chakoria Community Health Project (CCHP) of icddr,b, a low-performing sub-district in Cox’s Bazar district in Chittagong division, showed that only 27 of 48 scheduled SCs were held, thus poor use of SCs affected the CPR in the project area [17].

Although the present study was not intended to explore the non-programmatic factors for contraceptive use, previous works identified some important s non-programmatic factors such as age and education closely linked with the use of contraceptive methods. As per last BDHS 2007 report, CPR varied by different age groups; CPR was 42 percent among married women at age 15–19, while it ranged from 61–67 percent among women of age group 25 to 39 years [14]. It has been also reported by previous studies that use of contraceptive methods is associated with education [29]. Middle age group and better educated women were found to be motivated to accept contraceptive method [30]. Appropriate Information Education and Motivation (IEM) strategy may be developed for young and less educated women. Young and less educated women may be motivated on family planning methods through mothers clubs, and mobile help lines established for addressing needs of such groups of women.

A regular client-worker contact can improve the use of contraception. A policy of one worker per 150–200 eligible couples has been adopted in the icddr,b Matlab MCH-FP Project area to enhance the client-worker contact and to increase the use of contraception. A similar approach of low-paid community volunteer has been used in many countries, such as Nepal, Zimbabwe, Peru, India, Lagos, Nigeria, Latin America, and Columbia [31-36]. The objectives of these studies were to increase the access to family-planning services, oral rehydration therapy, and post-partum care and to increase the immunization coverage. A strategy to deploy low paid volunteers can be introduced particularly in hard-to-reach areas of Bangladesh. Public-private partnership may be strengthened to cover programmatic gaps and to improve family-planning performance, especially in low-performing areas. Non-former health care providers may be involved in the family planning program in reaching hard to reach unions. Community Clinics (CCs) (introduced to providing basic health and family planning services by Community Health Care Providers for 6,000 populations in village level) can be made functional in hard to reach unions to provide FP services.

There are some examples of innovative approaches to address needs of people in hard to reach areas, particularly for child immunization and maternal and neonatal care. For instance, a study done in Bangladesh to improve child immunization in hard to reach areas, offered a package of interventions and finally reported a higher rates of full immunization coverage at the end of the intervention period [37]. In Bangladesh, the first nationwide mobile phone health information service “Aponjon” under Mobile Alliance for Maternal Action (MAMA) has been started through United States Agency for International Development (USAID) in 2012 which is free for the poorest 20% of its subscribers [38]. The objective of the MAMA is to help pregnant women, new mothers and their families for pregnancy and delivery care. MAMA has already been carried good impact in South Africa, Indonesia and Bangladesh, where new mothers have access timely, and culturally relevant health information. Similar innovative interventions based on information technology (IT) should be piloted and introduced in FP programme in addressing FP needs of people in hard-to-reach areas of Bangladesh.

## Competing interests

The authors declare that they have no competing interests.

## Authors’ contributions

HK drafted the manuscript and was involved in field implementation and data analysis of the study. NC did statistical analysis. EO was involved in designing of the study and reviewed the manuscript. RG provided overall guidance to prepare the manuscript and acted as a mentor. All authors read and approved the final version of the manuscript.
